# Optimizing Bioremediation: Elucidating Copper Accumulation Mechanisms of *Acinetobacter* sp. IrC2 Isolated From an Industrial Waste Treatment Center

**DOI:** 10.3389/fmicb.2021.713812

**Published:** 2021-11-02

**Authors:** Wahyu Irawati, Eric Santoso Djojo, Lucia Kusumawati, Triwibowo Yuwono, Reinhard Pinontoan

**Affiliations:** ^1^Department of Biology Education, Universitas Pelita Harapan, Tangerang, Indonesia; ^2^Department of Biology, Universitas Pelita Harapan, Tangerang, Indonesia; ^3^Department of Food Technology, International University Liaison Indonesia, Tangerang, Indonesia; ^4^Department of Agricultural Microbiology, Universitas Gadjah Mada, Yogyakarta, Indonesia

**Keywords:** *Acinetobacter* sp. IrC2, bacteria, copper stress, lag phase, protein profile

## Abstract

*Acinetobacter* sp. IrC2 is a copper-resistant bacterium isolated from an industrial waste treatment center in Rungkut, Surabaya. Copper-resistant bacteria are known to accumulate copper inside the cells as a mechanism to adapt to a copper-contaminated environment. Periplasmic and membrane proteins CopA and CopB have been known to incorporate copper as a mechanism of copper resistance. In the present study, protein profile changes in *Acinetobacter* sp. IrC2 following exposure to copper stress were analyzed to elucidate the copper resistance mechanism. Bacteria were grown in a Luria Bertani agar medium with and without CuSO_4_ supplementation. Intracellular copper ion accumulation was quantified using atomic absorption spectrophotometry. Changes in protein profile were assessed using sodium dodecyl sulfate polyacrylamide gel electrophoresis. The results showed that 6 mM CuSO_4_ was toxic for *Acinetobacter* sp. IrC2, and as a response to this copper-stress condition, the lag phase was prolonged to 18 h. It was also found that the bacteria accumulated copper to a level of 508.01 mg/g of cells’ dry weight, marked by a change in colony color to green. The protein profile under copper stress was altered as evidenced by the appearance of five specific protein bands with molecular weights of 68.0, 60.5, 38.5, 24.0, and 20.5 kDa, suggesting the presence of CopA, multicopper oxidase (MCO), CopB, universal stress protein (Usp), and superoxide dismutase (SOD) and/or DNA-binding protein from starved cells, respectively. We proposed that the mechanism of bacterial resistance to copper involves CopA and CopB membrane proteins in binding Cu ions in the periplasm and excreting excess Cu ions as well as involving enzymes that play a role in the detoxification process, namely, SOD, MCO, and Usp to avoid cell damage under copper stress.

## Introduction

Heavy metal toxicity is the effect of heavy metals provoking bactericidal or bacteriostatic growth perturbation on bacteria. Such growth disturbance may occur depending on whether the bacteria have the capacity to absorb heavy metals (Arguello et al., 2013). Heavy metal exposure may result in the damage of cell membranes and a change in the specificity of enzymes by interfering with cellular function and by damaging the structures of DNA (Afzal et al., 2017). Copper is known as an important micronutrient used as a cofactor for enzymes such as cytochrome C oxidase. However, copper is also toxic as it causes lipid peroxidation, replacement of metal ions in proteins, formation of spurious disulfide bonds, and oxidation and degradation of iron–sulfur groups in proteins (Bondarczuk and Piotrowska-Seget, 2013). It has been suggested that cells develop a defense mechanism against copper toxicity while maintaining adequate intracellular concentrations of copper as a micronutrient (Rowland and Niederweis, 2013).

Cu (I) and Cu (II) can catalyze the production of reactive oxygen species (ROS) through the Fenton and Haber–Weiss reaction, which acts as a carrier to dissolve electrons that affect cytoplasmic molecules, DNA, lipids, and other proteins (Zhao et al., 2016). It is known that the Cu tolerance threshold varies among different bacteria. The detrimental effects of copper under extended copper stress may be alleviated by keeping the concentration of copper under the toxicity threshold inside the cells through active excretion of copper out of the cells or, alternatively, by accumulating copper inside the cells (Völlmecke et al., 2012; Bondarczuk and Piotrowska-Seget, 2013).

The proteins expressed by copper-induced genes play a role in bacterial copper homeostasis. This mechanism takes place in the bacterial cell during extended copper stress. The extended lag phase observed in a bacterial culture grown in media containing copper is possibly an adaptative response and/or resistance mechanism of bacteria to copper stress.

Therefore, changes in protein profile are expected during the lag phase (Rolfe et al., 2011). Proteins that play a role in the bacterial copper resistance mechanism enable bacteria to protect cell components from damage and allow normal cellular activities. The bacterial cell wall contains carbohydrates and phosphates that bind heavy metals (Yazid et al., 2012). During the adaptation process, the speed and direction of metabolic reactions will change in response to environmental stress. Metabolism is controlled by increasing and reducing protein synthesis as reflected in the protein profile (Tropp, 2012). It is known that copper increases the damage to DNA and proteins (Morris, 2014), which may result in nucleic acid structural changes and ultimately affect the protein synthesis processes, leading to metabolism disturbances (Fashola et al., 2016).

Proteins in the cell may be classified as structural and functional proteins. Structural proteins provide mechanical support, such as proteins in the cell wall, whereas functional proteins are biologically active proteins, such as enzymes (Nair and Finkel, 2004). A protein profile is a depiction of a protein synthesized under a given condition. Protein profile, protein sequence, and protein identity are important parameters in bacterial classification, identification, and physiology (Irawati et al., 2017a). The present study aims at understanding the effect of copper on the growth of *Acinetobacter* sp. IrC2, as well as obtaining the view of the bacterial mechanism of copper resistance through copper accumulation capability and protein profile changes following copper stress.

## Materials and Methods

### Medium for Bacterial Growth

*Acinetobacter* sp. IrC2 (accession number: JX009134) is a copper-resistant bacterium with a minimum inhibitory concentration of 10 mM CuSO_4_. *Acinetobacter* sp. IrC2 was cultivated in Luria Broth (LB; Pronadisa) containing 10 g/L tryptone, 5 g/L yeast extract, 10 g/L NaCl, and 0.1 g/L glucose for growth measurement. The medium was sterilized in an autoclave at 120°C for 15 min. A 1 M stock solution of CuSO_4_ (Merck) was prepared and filter-sterilized using a 0.2 μm syringe filter (Merck Millipore). Bacterial culture (0.5 ml, OD_600_ = 0.6) was inoculated into a 50 ml sterile LB medium. Bacterial cultures prepared with 6 mM copper and without copper supplementation were used as control, followed by shaking at 37°C and 100 rpm. Every 3 h, cell turbidity was measured using a spectrophotometer (LaboMed) at a wavelength of 600 nm until the bacteria reached the stationary growth phase. One milliliter of sample from each culture was withdrawn for OD_600_ measurement for each timepoint. Growth measurement was done in triplicates. The mean value of OD_600_ of cultures grown in media supplemented with CuSO_4_ was compared to that of the control group using Student’s t-test.

### Copper Accumulation in Media With Various Copper Concentration

Bacteria were grown in LB media containing 4, 5, 6, 7, 8, 9, and 10 mM CuSO_4_ and without CuSO_4_ as a control. Bacterial culture (0.5 ml) was inoculated into 25 ml of LB media, shaken at 37°C to stationary phase. Samples of cells were withdrawn periodically every 3 h for growth measurement, whereas the remaining cells were harvested by centrifugation at 5,000 × *g* for 20 min. Cells were oven-dried at 70°C for cell dry weight and total copper measurement. Cell dry weight was determined by using an analytical balance. Prior to copper content analysis, 50 ml of H_2_O was added to dried cells and shaken to homogenize the cell suspension. The sample solution was disrupted with HNO_3_ at 300°C until the solution became clear, followed by the addition of H_2_O to reach a 50 ml volume.

Intracellular copper content was determined by using atomic absorption spectrophotometry (AAS; Shimadzu AA 6800), at a wavelength of 324.9 nm. The AAS was prepared by installing a Cu hollow cathode lamp and burning acetylene gas (C_2_H_2_). The standard curve was made by measuring the Cu absorbance values of 0.2, 0.4, 0.6, 0.8, and 1.0 mg/L to obtain the equation of the regression line. Copper accumulation assay was performed in triplicates. The mean values of the assay were compared using Kruskal–Wallis one-way analysis of variance (Irawati et al., 2012b).

### Protein Profile Analysis

Ten milliliters of bacterial cultures were harvested at the log and stationary phases by centrifugation at 5,000 × *g* for 20 min, followed by washing and resuspending the cells in 1 ml of phosphate buffer pH 7. The cell suspension was then frozen at −70°C before being thawed at room temperature and sonicated for 5 min at 4°C. The cell fragments were separated by centrifugation at 16,278 × *g* for 10 min at 4°C to obtain an intracellular supernatant/protein. The intracellular proteins were separated by using the sodium dodecyl sulfate polyacrylamide gel electrophoresis (SDS-PAGE) method with a constant electric current of 25 mA until bromophenol blue of the protein ladder approached the end of the resolving gel. The concentration of acrylamide in the stacking gel was 4%, and the resolving gel was 12% with a gel thickness of 1.5 mm (Patricia and Irawati, 2017).

The stacking gel consisted of 4% bis-acrylamide, 1 M Tris–HCl pH 6.8, 10% SDS, tetrametilendiamine, and 1.5% ammonium persulfate. Similarly, the resolving gel was made from the same ingredients except that the 1 M Tris–HCl was prepared at pH 8.6. The intracellular protein was visualized by Coomassie blue staining, soaked for 30 min, and washed with a destaining solution. The molecular weight of the protein profile was determined based on the standard curve of molecular weight from the protein ladder (Thermo Fisher Scientific, cat. number 26610) composed of beta-galactosidase (116 kDa), bovine serum albumin (66.2 kDa), ovalbumin (45.0 kDa), lactate dehydrogenase (35.0 kDa), REase Bsp98I (25.0 kDfa), beta-lactoglobulin (18.4 kDa), and lysozyme (14.4 kDa) with storage buffer containing bromophenol blue.

## Results and Discussion

### Impact of Copper on the Growth of *Acinetobacter* sp. IrC2 at Different Concentrations

**FIGURE 1:**
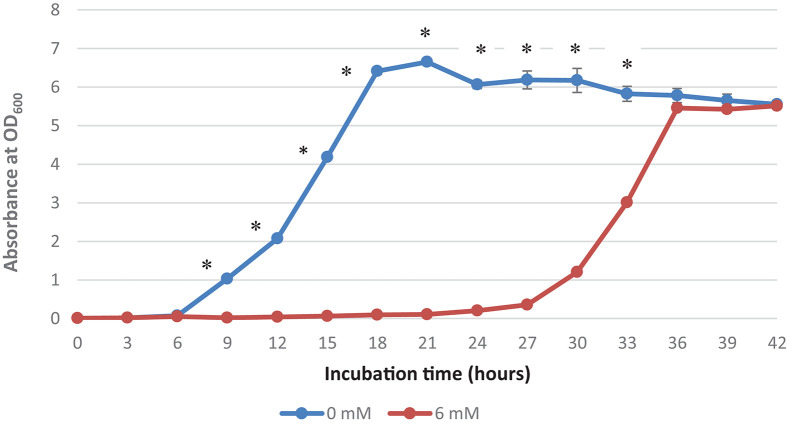
Growth curve of *Acinetobacter* sp. IrC2. Growth curve of *Acinetobacter* sp. IrC2 culture grown in LB media added with two different CuSO_4_ concentrations, i.e., 0 mM and 6 mM. One ml of sample from each culture was taken for OD measurement for each timepoint. Optical Density (OD) measured at a wavelength of 600 nm is 0.6 Triplicate measurements were done for each time point. Error bars represent standard deviations. * Represents *P* value < 0.05.

It was observed that the addition of 6 mM copper sulfate in the growth medium prolonged the lag phase to 18 h, while no lag phase was observed in the control treatment (Figure 1). A high concentration of copper has been reported to affect the metabolism and growth of bacteria as well as other bacterial activities, thereby resulting in a prolonged lag phase and even death (Benhalima et al., 2020). This extended lag phase indicates copper stress and the adaptation to this environmental stress by the surviving bacteria (Rolfe et al., 2011). Bertrand (2019) has termed this phenomenon “tolerance by lag” as prolonged lag phases are adaptive responses to stress and injury that contribute to the ability of bacteria to confer copper resistance.

Mathews et al. (2013) suggested that copper-bacterial contact resulted in the damage of the cell envelope, which consequently makes the cells more susceptible to damage by copper ions. When bacterial cells are exposed to copper, they experience a decrease in the number of viable cells and hence require more time for physiological recuperation before exiting the lag phase and undergoing exponential growth (Dupont and Augustin, 2009). Extended lag phases allow the bacteria to induce DNA repair mechanisms to repair and replace damaged cellular components due to exposure to copper and oxidative stress as well as initiate a novel transcriptional program that transforms their transcriptome and proteome to produce genes and proteins needed for metabolic processes, biomass accumulation, and cell division (Larsen et al., 2006). Watanabe et al. (2015) also suggested that bacteria maintain their growth, development, and survival by altering their enzymatic profiles during the prolonged lag phase in response to various nutrients and contaminants, including copper.

### Copper Toxicity in *Acinetobacter* sp. IrC2

**FIGURE 2:**
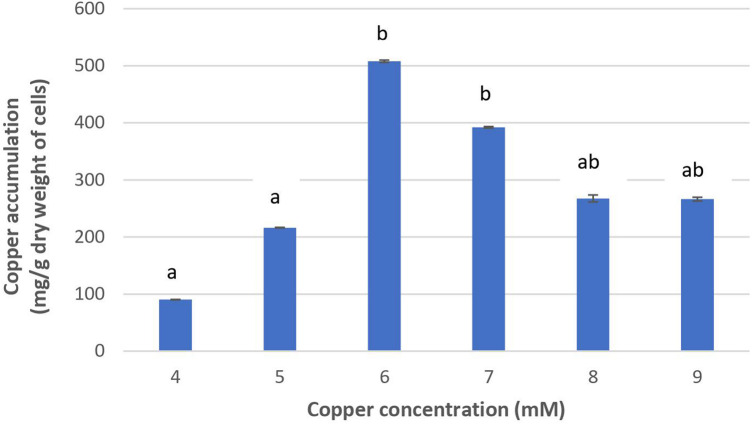
The copper accumulation ability of *Acinetobacter* sp. IrC2. Values of each treatment group are the mean of triplicates measurement. Error bars represent standard deviations. Different letters indicate significant differences between groups (*P* value <0.05).

Figure 2 shows that the increase of copper concentration (4–6 mM) resulted in the increase of copper accumulation in cells. However, copper concentration higher than 6 mM resulted in reduced copper accumulation. The highest level of copper concentration (508.01 mg/g cells) was achieved at 6 mM. These results suggest that *Acinetobacter* sp. IrC2 managed copper stress by accumulating copper in the cells. When the cells were exposed to copper concentrations higher than 6 mM, intracellular copper accumulation was found reduced. Such reduction suggested that copper toxicity increased with the increase of copper concentration of more than 6 mM, which resulted in the restriction of bacterial tolerance to copper.

Increased copper toxicity affects the physiological properties of bacteria by causing oxidative damage and the reduction of essential nutrients absorption; hence, the bacteria are unable to metabolize and synthesize the necessary adsorptive agents to accumulate copper (Mateos-Naranjo et al., 2013). Furthermore, copper toxicity may block functional groups on the cell surface, the substitution of essential metal ions through biomolecular modifications, denaturation, and inactivation of enzymes, along with the disruption of cellular and organellar membrane integrity (Gadd, 1993). Another possibility is that copper-binding sites on the bacterial cell membrane lack both the availability and capacity to accumulate copper at higher concentrations, most likely due to exhaustion from an extended overexposure to copper (Irawati et al., 2017b).

Timkova et al. (2018) defined bioaccumulation as the absorption of toxic pollutants that enter actively into the cell across the cell membrane and accumulate intracellularly. The level of toxic pollutants’ accumulation depends on the intrinsic structural and biochemical properties, genetic and physiological adaptations, modification of the metal environment, and availability and toxicity of metals. Intracellular copper uptake may be achieved through a variety of biochemical mechanisms (Rehman et al., 2008), which involve the synthesis of adsorptive agents, especially proteins during their lag phase. These agents bind copper ions onto the cell surface before they are actively transported across the cell membrane and into the cytoplasm or periplasm where they are accumulated and stored (Timkova et al., 2018). It is also important to note that metal accumulation is influenced by the surface characteristics, such as surface charges of the microorganism. Gram-negative bacteria are equipped with negatively charged functional groups (such as carboxyl, hydroxyl, phosphoryl, and amide groups) on their surface; hence, they are capable of attracting and binding positively charged cationic metals *via* counter-ion interactions (Jiang et al., 2004; Spain et al., 2021). Successfully bound copper ions will also be intracellularly accumulated and stored, as often evidenced by a morphological change in colony color.

A previous study reported that in adapting to high copper stress, *Acinetobacter* sp. IrC2 absorbed copper as part of its tolerance mechanism. The color of bacterial colonies grown on agar media supplemented with 5 mM CuSO_4_ turned to green as a result of copper absorption (Irawati et al., 2012b). Similarly, the colony of yeast isolates obtained from the Surabaya wastewater treatment also changed to green when accumulating copper (Irawati et al., 2012a).

### Protein Profile Analysis of *Acinetobacter* sp. IrC2

**FIGURE 3:**
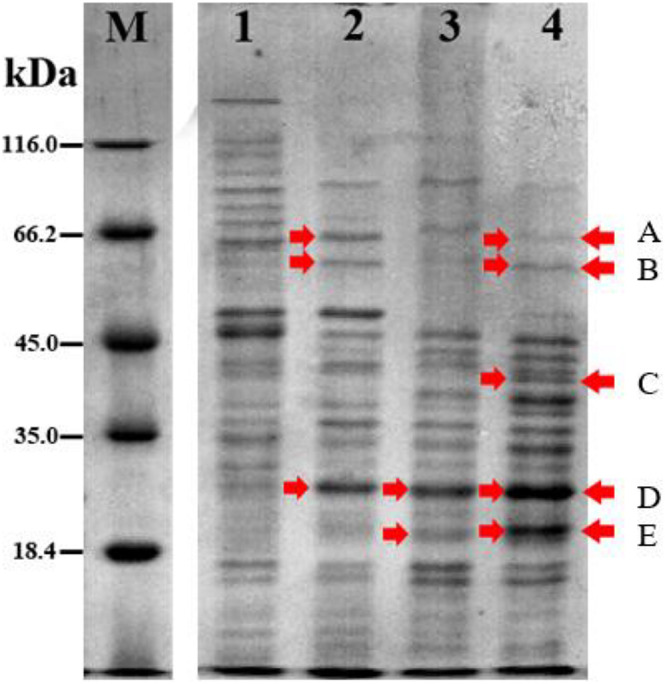
Protein profile of *Acinetobacter sp.* IrC2 during log phase and stationary phase in media with 5 mM CuSO_4_ and without CuSO_4_. (M) Protein Ladder; (1) log phase in LB media without addition of CuSO_4_; (2) log phase in LB media with addition of CuSO_4_; (3) stationary phase in LB media without addition of CuSO_4_; (4) stationary phase in LB media with addition of CuSO_4_. Red arrow indicates proteins that are synthesized more after copper sulfate induction.

The protein profile of *Acinetobacter* sp. IrC2 (Figure 3) showed the increased synthesis of at least five protein bands after copper induction, i.e., protein band A (68.0 kDa), protein band B (60.5 kDa), protein band C (38.5 kDa), protein band D (24.0 kDa), and protein band E (20.5 kDa), which might include CopA, multicopper oxidase (MCO), CopB, universal stress protein (Usp), and superoxide dismutase (SOD) and/or DNA-binding protein from starved cells, respectively (Table 1). Protein profiles of *Acinetobacter* sp. IrC2 showed differences in protein profiles between bacteria grown under copper stress and without stress, observed at the log and stationary phases (Figure 3). Several proteins detected at the log phase were absent when the cells entered the stationary phase. However, new proteins were detected at the stationary phase. The changes of protein profiles at the log and stationary phases when the cells were not under copper stress may be attributed to nutrition depletion at the stationary phase. Differences in protein profile under copper stress and no stress at the stationary phase suggested that the protein synthesized at the stationary phase plays a role in the copper resistance mechanism. A similar phenomenon was also observed in an HgP1 isolate, that is, a mercury-resistant bacterium isolated from a gold mine in Pongkor (Patricia and Irawati, 2017). The protein profile of HgP1 showed that the proteins synthesized under normal conditions were less than those synthesized under mercury stress. Moreover, the synthesis of protein, presumed to play a role in the mercury resistance mechanism, was found to increase under mercury stress. Bacterial cells alter the transcription pattern of a specific set of genes by reducing normal protein synthesis and synthesizing a specific set of proteins in response to mercury toxicity. This specific set of proteins include a heat-shock protein or stress protein.

**TABLE 1:** Proteins synthesized at higher level after copper sulfate induction in *Acinetobacter sp*. IrC2.

**Protein band**	**Molecule weight (kDa)**	**Prediction of containing protein**	**References**
A	68.0	Copper resistance protein A (CopA)	Harkins et al. (2013)
B	60.5	Multicopper oxidase (MCO)	Singh et al. (2013)
C	38.5	Copper resistance protein B (CopB)	Cha and Cooksey (1991)
D	24.0	Universal stress protein (Usp)	Chi et al. (2019)
E	20.5	Superoxide dismutase (SOD)	Harkins et al. (2013)

*Proteins prediction based on molecular weight and increase in protein synthesis after copper sulfate induction.*

It was observed that proteins synthesized by *Acinetobacter* sp. IrC2 can be grouped into three different levels of synthesis and functions, i.e., (1) constitutively synthesized proteins under both normal conditions and copper stress, (2) less or nonsynthesized proteins under copper stress, and (3) proteins that are synthesized at a higher level under copper stress. Cvjetko et al. (2014) stated that heavy metals can induce changes in gene expression.

The constitutively synthesized proteins under normal and copper-stress conditions indicate their importance for the basic growth metabolism of the cell. By contrast, the less or nonsynthesized proteins under copper stress suggest the alteration of gene expression patterns as a consequence of copper stress, similar to that in heat-shock proteins synthesized under heat-shock stimuli. Heat-shock proteins are now understood to perform critical functions to the cell under stress and no stress conditions (Miller and Fort, 2018). In addition, copper-stress stimuli may also induce damage to enzymatic functions and disruption of ion regulation implicated in the disruption of DNA and protein synthesized (Gauthier et al., 2014).

The upregulated proteins under copper stress indicated their role in response to copper toxicity. Staehlin et al. (2016) suggested that heavy metal exposure induces changes in protein synthesis, particularly for proteins involved in the stress resistance mechanism.

Ayudhya et al. (2009) reported that heavy metal toxicity causes bacteria to carry out four mechanisms: (i) increase the synthesis of several enzymes involved in catabolic regulation to maintain cellular energy production; (ii) initiate the use of low-energy transport systems to import amino acids and other carbon sources; (iii) facilitate the synthesis of metal-binding amino acids; and (iv) enhance the metal transport system for intracellular metal regulation as a form of adaptation.

These results were similar to those reported by Gillan et al. (2017), who suggested that bacteria can synthesize 19 specific protein groups depending on the concentration and exposure of Cu (II). These specific proteins are expressed in response to high concentrations of Cu (II) involved in periplasmic and cytoplasmic detoxification.

The protein profile determined by the present study showed that a specific protein with a molecular weight of 68.0 kDa was only synthesized under copper induction, thus indicating that this protein may have a role in bacterial resistance to copper. Based on its molecular weight, it is suggested that protein band A (Table 1) contained copper resistance protein A (CopA). This assumption is also supported by the fact that the size of the CopA protein in *Acinetobacter* is 64.5–72.5 kDa (Harkins et al., 2013). Williams et al. (2016) suggested that copper impairs the integrity of the outer membrane, generates radicals, inhibits respiration, and degrades DNA. Alquethamy et al. (2019) reported that the P-type CopA ATPase is a major contributor to *Acinetobacter baumannii* and *Legionella pneumophila* to export copper from the cytoplasm as a self-defense mechanism against copper toxicity and protection against oxidative stress.

It is presumed that *Acinetobacter* sp. IrC2 synthesized the CopA protein during the lag phase for 18 h in a copper-supplemented medium to protect cells from toxicity by accumulating copper, as evidenced by the color change of colonies to green (Figure 3). Irawati et al. (2012b) reported that *Acinetobacter* sp. IrC2 is a copper-resistant bacteria isolated from the waste treatment center in Surabaya that accumulates copper to adapt to its toxic environment. The absorbed copper ions (Cu^2+^) bound to the CopA protein resulted in the color change of the colony to blue (Irawati et al., 2012a). Copper stress induces *copA* gene expression in bacteria that results in the alteration of protein expression.

Similar studies (Irawati et al., 2016) also reported that *Pseudomonas syringae* and *Acinetobacter* sp. IrC1 harbor *copA* gene responsible for bacterial resistance to copper by bioaccumulating copper in cells and color change of bacterial colonies to blue. *Pseudomonas syringae* has copper resistance genes, *cop* gene operons that are regulated and induced only by high levels of copper (Behlau et al., 2011). These genes help express copper-binding proteins including CopA and CopB that take part in accumulating copper inside the periplasm to prevent toxic levels of copper in the cytoplasm. Shahla et al. (2014) stated that the CopA protein in *P. syringae* plays a role in binding excess copper ions in either the periplasm or the cytoplasm and translocating them to the periplasm. CopA and CopB are periplasmic binding protein and outer membrane protein, respectively, required to support copper resistance mechanisms. Copper resistance is mediated by sequestration of copper in the periplasm by the copper-binding proteins CopA and CopC and in the outer membrane by CopB.

The protein profile determined by the present study also shows that a specific protein band with a molecular weight of 60.5 kDa had increased synthesis after copper induction in bacteria. Based on its molecular weight, this protein band possibly contained the MCO family. MCO is an enzyme that contains four copper units and undergoes increased synthesis when bacteria are grown in a medium containing copper (Singh et al., 2013). The molecular weight range of MCO in the genus *Acinetobacter* is 49.4–67.3 kDa. Wen et al. (2014) reported that MCO is required for copper resistance in some bacterial species. MCO is a part of the bacterial membrane and plays a role in the resistance and detoxification mechanism of copper by oxidizing toxic Cu^2+^ ions in the periplasm (Rowland and Niederweis, 2013). It is therefore suggested that protein band B (Table 1) contained an MCO.

Free copper is dangerous owing to its high chemical reactivity (Arguello et al., 2013). Therefore, bacteria must control the transport of Cu^2+^ ions through the compartments to ensure copper homeostasis in cells and prevent toxicity. Copper binding with cysteine in the membrane produces a blue color as a characteristic of MCO activity (Rowland and Niederweis, 2013). These facts thus supported the observation that *Acinetobacter* sp. IrC2 extended the lag phase for 18 h by synthesizing MCO as a resistance mechanism. Such response was manifested in the appearance of blue colonies when bacteria were grown on a 6 mM CuSO_4_ medium.

The 1D SDS-PAGE also revealed a protein band with a molecular weight of 38.5 kDa synthesized during the stationary phase in cells grown in media supplemented with copper sulfate. Such protein, however, was not detected during the log phase. This specific protein band’s molecular weight (38.5 kDa) is close to the molecular weight of CopB (39 kDa), as suggested by Cha and Cooksey, 1991. Therefore, it is suggested that protein band C (Table 1) carried a CopB protein. CopB is a peripheral membrane protein located on the outer membrane, which can be released into the periplasm when the cell changes to the spheroplast. CopB is a Cu^2+^ATPase with a role in mediating the efflux of cytoplasmic Cu^+/2+^ (Padilla-Benavides et al., 2013; Raimunda et al., 2013; Gillan et al., 2017; Thummeepak et al., 2020), as CopB is a protein located on the outer membrane required to support copper resistance mechanisms.

At log and stationary phases, a protein band with a molecular weight of 24.0 kDa was detected when *Acinetobacter* sp. IrC2 was grown in LB media supplemented with CuSO_4_. It is proposed that this protein band contained protein D that belongs to the Usp family. The Usp family in the genus *Acinetobacter* is known to have a molecular weight of 12.7–31.2 kDa (Snitkin et al., 2013). Usp is synthesized when bacteria are under various kinds of stress, such as nutrient deficiencies, heavy metals, and oxidative stress. Copper can catalyze the production of ROS, which can cause oxidative stress. Usp has a role in oxidative stress resistance; therefore, it is proposed that Usp has an indirect role in copper-stress resistance. Usp acts to prevent denaturation of macromolecules and repair and protect its nucleic acids from external stress (Chi et al., 2019).

During the stationary phase, a protein band with a molecular weight of 20.5 kDa was detected in *Acinetobacter* sp. IrC2 cells were cultivated under both copper stress and no copper stress conditions. It is proposed that, based on its molecular weight, this protein band (designated as protein band E, Table 1) contained an SOD protein and/or protein that belongs to the DNA-binding protein family from starved cells (Dps). Jung et al. (2010) suggested that SOD in the genus *Acinetobacter* has a molecular weight between 19.4 and 21.0 kDa.

It has been suggested that copper can catalyze the production of ROS, such as superoxide, which in turn can cause oxidative stress (Harkins et al., 2013). SOD is a metalloenzyme that converts highly toxic superoxide into oxygen and less toxic hydrogen peroxide. Therefore, proper evaluation of the bioremediation efficiency of a given bacterial strain should be determined based on its physiological response to heavy metals. SOD is also known to catalyze the dismutation of superoxide into oxygen and hydrogen peroxide. Hydrogen peroxide is a strong oxidant and has to be degraded by catalase into H_2_O and O_2_. SOD protects DNA from oxidative stress and is produced by a broad range of bacteria, especially in the lag phase where bacteria cells are under nutrient deficiency stress.

### Mechanism of Copper Resistance in *Acinetobacter* sp. IrC2 Based on Protein Profile

The results of this study suggested that *Acinetobacter* sp. IrC2 synthesizes five proteins during copper induction, namely, CopA, CopB, MCO, Usp, and SOD. We proposed that these proteins are involved in the acquisition of resistance mechanisms to copper in bacteria.

Copper is one of the important elements required by bacteria for metabolic processes; however, it is highly toxic when it is above the bacterial tolerance threshold, so bacteria must be able to control Cu homeostasis in cells (Zhao et al., 2016). Ji and Silver (1995) suggested that the *cop* operon encodes periplasmic proteins (CopA and CopC), outer membrane proteins (CopB), and inner membrane proteins (CopD), induced by the presence of copper. Copper is bound by the CopB protein and transported into the periplasm. In the periplasm, copper is bound by the protein CopA. CopC and CopD proteins are also facilitated in the influx of copper from the periplasmic space into the cytoplasm for transfer to enzymes that require it as a cofactor.

Cu homeostasis is controlled by the CopB protein, which mediates transportation of Cu into the cells and binds Cu to specific sites to act as an enzyme cofactor (Kaplan and Maryon, 2016). CopB also plays a role in regulating the release of excess Cu^+/2+^ ions. Cu^2+^ ions will be bound by the CopA protein in the periplasm to block the entry of excess copper ions into the cytoplasm (Shahla et al., 2014). MCO is a part of the bacterial membrane that plays a role in the resistance mechanism and detoxification of copper by oxidizing toxic Cu^+^ ions to Cu^2+^ in the periplasm (Alquethamy et al., 2019). Free copper has a high chemical reactivity, which necessitates the bacteria to control Cu^+^ ions by transporting them out of cells through the CopB protein to ensure copper homeostasis in cells and prevent toxicity. MCO synthesis as a resistance mechanism resulted in the blue color change of colonies on *Acinetobacter* sp. IrC2.

Inside the cell, Cu ions will undergo an oxidation–reduction reaction that may result in the excess of Cu^+^ and Cu^2+^ ions in the cell. Cu ions may act as strong inhibitors of cellular enzymes and react freely with various protein side chains containing sulfur, nitrogen, and oxygen (Kaplan and Maryon, 2016). Cu^+^ and Cu^2+^ catalyze the production of ROS, which results in the damage of molecules, DNA, lipids, and proteins (Zhao et al., 2016).

The production of ROS such as superoxide, hydroxy radicals, and hydrogen peroxide in cells causes oxidative stress (Kong et al., 2020). SOD is a metalloenzyme synthesized in the lag phase that acts as the main antioxidant and is responsible for protecting the DNA from ROS. SOD plays a role in converting highly toxic superoxide into less toxic oxygen and hydrogen peroxide. Hydrogen peroxide is a strong oxidant and must be degraded by catalase into H_2_O and O_2_ (Harkins et al., 2013; Kong et al., 2020).

The Usp family has a role in oxidative stress resistance under copper-stress state. It acts to prevent macromolecular denaturation, as well as to repair and protect nucleic acids from external stresses (Chi et al., 2019). It also plays a role in regulating the capacity of cells to withstand oxidative agents and helps the cells to resist the presence of H_2_O_2_ during growth (Nachin et al., 2005).

## Conclusion

This study showed that the addition of 5 mM CuSO_4_ into growth media resulted in copper stress to *Acinetobacter* sp. IrC2, which imposed a physiological response by extending the lag phase for 18 h. It was also found that the cells developed a resistance mechanism to copper by accumulating copper in the cells. Five protein bands with molecular weights of 68.0, 60.5, 38.5, 24.0, and 20.5 kDa were detected under copper stress. It is proposed that those protein bands contained important proteins involved in the bacterial resistance mechanism during copper stress. Therefore, it is suggested that bacterial resistance to copper is mediated by CopA and CopB membrane proteins in binding Cu ions in the periplasm and excreting excess Cu ions. In addition, other enzymes that play a role in the detoxification process, namely, SOD, MCO, and Usp, may also act in concert to avoid cell damage under copper stress.

It is of concern that identifying the proteins will add more depth of understanding regarding the physiological shift when the bacterium was subjected to heavy metal stress. However, such identification process requires a different approach of research, which means a new work following this study. The current work was aimed at demonstrating that heavy metal stress altered the physiological response of the bacteria, as evidenced by the synthesis of several new proteins, as well as proposing the underlying mechanism of the stress response. It is anticipated that proteome and transcriptome analysis will provide more details in the elucidation of heavy metal resistance, particularly Cu, in bacteria so as to give a better understanding on global metabolism.

## Data Availability Statement

The original contributions presented in the study are included in the article/Supplementary Material, further inquiries can be directed to the corresponding author/s.

## Author Contributions

WI was the group leader, who designed the study, carried out laboratory works, analyzed the data with ED and LK, and prepared the article draft. LK conducted part of the laboratory works, analyzed the data, and edited the final manuscript. RP contributed to the writing of the article draft. TY was responsible for finalizing the article for publication. All authors contributed to the article and approved the submitted version.

## Conflict of Interest

The authors declare that the research was conducted in the absence of any commercial or financial relationships that could be construed as a potential conflict of interest.

## Publisher’s Note

All claims expressed in this article are solely those of the authors and do not necessarily represent those of their affiliated organizations, or those of the publisher, the editors and the reviewers. Any product that may be evaluated in this article, or claim that may be made by its manufacturer, is not guaranteed or endorsed by the publisher.
