# Supplementary material for: Optimizing Bioremediation: Elucidating Copper Accumulation Mechanisms of Acinetobacter sp. IrC2 Isolated From an Industrial Waste Treatment Center
Source: Front Microbiol. 2021 Nov 2;12:713812. doi: 10.3389/fmicb.2021.713812 (PMC8595058; doi:10.3389/fmicb.2021.713812)
Supplement: Supplementary file 1 [file Data_Sheet_1.pdf]

Supplementary Materials

1. Comparison between Growth Curve of Control Culture and Growth Curve of Culture with 5 mM $CuSO_4$

Replication	Time point (hours after inoculation)	OD_{600} of culture with 0 mM $CuSO_4$	Mean	SD	OD_{600} of culture with 5 mM $CuSO_4$	Mean	SD	Pvalue
1	0	0.007	0.007	0.000	0.007	0.006	0.001	0.130
2	0	0.007			0.005			
3	0	0.007			0.005			
1	3	0.011	0.016	0.005	0.019	0.016	0.004	0.935
2	3	0.015			0.018			
3	3	0.021			0.011			
1	6	0.076	0.075	0.006	0.023	0.047	0.045	0.390
2	6	0.081			0.019			
3	6	0.069			0.099			
1	9	1.011	1.031	0.027	0.023	0.021	0.002	0.000
2	9	1.021			0.021			
3	9	1.062			0.020			
1	12	2.023	2.076	0.046	0.046	0.039	0.006	0.000
2	12	2.098			0.037			
3	12	2.107			0.034			
1	15	4.177	4.186	0.048	0.071	0.064	0.009	0.000
2	15	4.143			0.054			
3	15	4.237			0.068			
1	18	6.45	6.407	0.080	0.110	0.096	0.015	0.000
2	18	6.457			0.098			
3	18	6.315			0.081			
1	21	6.733	6.648	0.075	0.117	0.106	0.010	0.000
2	21	6.618			0.102			
3	21	6.593			0.099			
1	24	6.05	6.065	0.104	0.211	0.204	0.006	0.000
2	24	6.175			0.201			
3	24	5.969			0.199			
1	27	6.36	6.184	0.232	0.373	0.356	0.017	0.000
2	27	6.271			0.356			
3	27	5.921			0.340			
1	30	6.5	6.170	0.312	1.203	1.201	0.002	0.001
2	30	6.132			1.201			
3	30	5.879			1.199			

1	33	5.61	5.824	0.194	3.017	3.010	0.007	0.002
2	33	5.99			3.010			
3	33	5.871			3.004			
1	36	5.63	5.778	0.182	5.460	5.456	0.006	0.092
2	36	5.982			5.458			
3	36	5.723			5.449			
1	39	5.47	5.653	0.161	5.425	5.419	0.007	0.129
2	39	5.776			5.421			
3	39	5.712			5.412			
1	42	5.53	5.547	0.068	5.570	5.512	0.089	0.614
2	42	5.622			5.556			
3	42	5.49			5.410			

**Time point (hours after inoculation)**	**Mean of OD_{600} of culture with 0 mM $CuSO_4$**	**Standard Deviation**	**Mean of OD_{600} of culture with 5 mM $CuSO_4$**	**Standard Deviation**	**Pvalue**
0	0.007	0.000	0.006	0.001	0.130
3	0.016	0.005	0.016	0.004	0.935
6	0.075	0.006	0.047	0.045	0.390
9	1.031	0.027	0.021	0.002	0.000*
12	2.076	0.046	0.039	0.006	0.000*
15	4.186	0.048	0.064	0.009	0.000*
18	6.407	0.080	0.096	0.015	0.000*
21	6.648	0.075	0.106	0.010	0.000*
24	6.065	0.104	0.204	0.006	0.000*
27	6.184	0.232	0.356	0.017	0.000*
30	6.170	0.312	1.201	0.002	0.001*
33	5.824	0.194	3.010	0.007	0.002*
36	5.778	0.182	5.456	0.006	0.092
39	5.653	0.161	5.419	0.007	0.129
42	5.547	0.068	5.512	0.089	0.614

Growth measurement was done in triplicates for both cultures grown with 5 mM $CuSO_4$ and control. The mean value of OD_{600} of cultures grown in media added with $CuSO_4$ was compared to that of the control group using the Student’s t test. * represents Pvalue< 0.05. Student’s t test was performed using Microsoft Excel 2019.

2. Cu Accumulation of bacteria cells grown in media added with different $CuSO_4$ concentration

$CuSO_4$ concentration (mM)	Mean of accumulated Cu (mg/g dry weight of cells)	Standard Deviation
4	90.187	0.413
5	216.333	0.597
6	508.003	2.000
7	392.160	1.353
8	267.517	6.113
9	266.307	3.321

Test Statistics[a,b]

	accumulated _Cu
Kruskal-Wallis H	16.111
df	5
Asymp. Sig.	.007

a. Kruskal Wallis Test

b. Grouping Variable: concentration

Multiple Comparison (Least Significant Difference)

(I) Concentration	(J) Concentration	Mean Difference (I-J)	Pvalue
4 mM	5 mM	-126.147*	0.000
	6 mM	-417.817*	0.000
	7 mM	-301.973*	0.000
	8 mM	-177.330*	0.000
	9 mM	-176.120*	0.000
5 mM	4 mM	126.147*	0.000
	6 mM	-291.670*	0.000
	7 mM	-175.827*	0.000
	8 mM	-51.183*	0.000
	9 mM	-49.973*	0.000
6 mM	4 mM	417.817*	0.000
	5 mM	291.670*	0.000
	7 mM	115.843*	0.000
	8 mM	240.487*	0.000
	9 mM	241.697*	0.000
7 mM	4 mM	301.973*	0.000
	5 mM	175.827*	0.000
	6 mM	-115.843*	0.000
	8 mM	124.643*	0.000

	9 mM	125.853*	0.000
8 mM	4 mM	177.330*	0.000
	5 mM	51.183*	0.000
	6 mM	-240.487*	0.000
	7 mM	-124.643*	0.000
	9 mM	1.210	0.633
9 mM	4 mM	176.120*	0.000
	5 mM	49.973*	0.000
	6 mM	-241.697*	0.000
	7 mM	-125.853*	0.000
	8 mM	-1.210	0.633

* The mean difference is significant at the 0.05 level.
